# A Reference chart for clinical biochemical tests of hemolyzed serum samples

**DOI:** 10.1002/jcla.23561

**Published:** 2020-09-02

**Authors:** Jun Ni, Wenbo Zhu, Yanyang Wang, Xuefei Wei, Jingjing Li, Lu Peng, Kui Zhang, Bing Bai

**Affiliations:** ^1^ Department of Laboratory Medicine Nanjing Drum Tower Hospital Clinical College of Nanjing Medical University Nanjing China; ^2^ Department of Nuclear Medicine Nanjing Drum Tower Hospital The Affiliated Hospital of Nanjing University Medical School Nanjing China; ^3^ Center for Precision Medicine Nanjing Drum Tower Hospital The Affiliated Hospital of Nanjing University Medical School Nanjing China; ^4^ Department of Laboratory Medicine Affiliated Nanjing Brain Hospital Nanjing Medical University Nanjing China

**Keywords:** clinical biochemical test, hemogram test, hemolysis, hemolytic index

## Abstract

**Background:**

Although the effect of hemolysis has been extensively evaluated on clinical biochemical tests, a practical guidance for laboratory staff to rapidly determine whether a hemolyzed blood sample is acceptable and how to interpret the results is lacking. Here, we introduce a chart as a convenient reference for dealing with such samples.

**Methods:**

Serum samples with 0.1%, 0.3%, 1%, 3%, and 10% hemolysis were prepared from sonicated endogenous red blood cells and received 35 wet and 22 dry clinical biochemical tests, respectively. The contributing part in the biochemical test result at each hemolysis condition was derived by subtracting the original test result of this sample with no hemolysis. The net results were used for analyses and preparation of the reference chart.

**Results:**

The reference chart displayed the analytically calculated hemolysis interference and related statistical analyses. The chart also provided the color appearance of serum samples at each hemolysis condition for clinical staffs to determine whether a hemolyzed sample could be accepted.

**Conclusion:**

In clinical laboratories, preparation of such a reference chart is extremely useful in dealing with hemolyzed blood samples for clinical biochemical tests.

## 1 INTRODUCTION

In clinical laboratories, hemolysis is one of the major problems that interfere with biochemical tests,^1^, ^2^, ^3^ representing a frequent reason for blood sample rejection.^4^, ^5^, ^6^ For example, the normal range of serum potassium ion (K^+^) concentration is 3.5 ~ 5.5 mmol/L, while the intracellular potassium ion (K^+^) concentration is approximately 150 mmol/L. Therefore, even 1% of red blood cell hemolysis in the serum will add 1.5 mmol/L to the K^+^ result, yielding a cautious K^+^ value that might initiate unnecessary medical actions. Indeed, it is commonly known that K^+^ measurement is affected by hemolysis. Similar problems also occur with measurements of lactate dehydrogenase (LDH) and creatine kinase‐MB (CK‐MB), which are critical for the diagnosis of emergent cardiac events.

Hemolyzed samples are often rejected for some routine biochemical. However, this is problematic when patients are in unfavorable conditions, and redrawing a blood sample is not feasible, or when hemolysis is an actual medical condition in some patients.^7^, ^8^

The impact of hemolysis on clinical chemistry tests has been extensively investigated,^9^, ^10^, ^11^ and quantitative analyses have been provided in numerous reports.^2^, ^3^, ^12^ Additionally, the appropriate management of these samples in clinical laboratories has been suggested.^13^, ^14^, ^15^ However, handy practical guidance as a convenient reference for clinical technicians to ascertain whether a hemolyzed sample for routine biochemistry testing is acceptable and how to interpret the results of these samples after analyses is urgently needed. Therefore, we performed this study to provide a chart that can be easily used as a reference by clinical laboratories when dealing with hemolyzed blood samples during routine clinical biochemistry tests.

## 2 MATERIALS AND METHODS

### 2.1 Blood sample collection and hemolysis preparation

Blood samples were collected from individuals during a routine physical examination for health screening at our clinical laboratory. Samples with an increase in the white blood cell count, positive HBsAg, or any other signs of infectious conditions were excluded. All samples were further screened for HIV, HCV, and syphilis antibodies, and the results were negative. For the serum samples, the biochemical test results covered the relatively low, moderate, and high range as much as possible. Additionally, EDTA‐anticoagulated whole blood samples (used for the routine blood cell count test) were collected from the same individual in a sufficient amount for artificial hemolysis preparation. This ensured the condition of endogenous hemolysis and avoided a possible aggregation resulting from unmatched blood types. Finally, 20 serum samples, with corresponding EDTA‐whole blood samples, were collected.

Briefly, 1.0 mL of the EDTA‐anticoagulated whole blood samples was centrifuged to retain the red blood cells (RBCs) and then washed with saline three times until the saline became clear. Deionized water was added to the RBCs to achieve a volume of 1.0 mL. As this was insufficient to break down RBCs completely, the sample was then sonicated by a microtip at 30% amplitude for 5 seconds three times, at 5‐second intervals. Complete hemolysis was confirmed under a microscope. The same sonication condition was applied to a serum sample in a preliminary study to ensure that the sonication imposed no impact on the clinical biochemistry analytes because sonication might denature the biochemical enzymes, generating false‐negative results. Next, 0.1, 0.03, and 0.01 mL of the above prepared hemolyzed sample were added to a 0.9, 0.97, and 0.99 mL serum, respectively, to achieve 10%, 3%, and 1% hemolyzed samples. Additionally, 0.3% and 0.1% hemolyzed serum samples were prepared from the 3% and 1% hemolysis samples. Hemoglobin concentrations in the samples were measured using OC‐SENSOR IO (Eiken Chemical Co., Ltd.) with appropriate dilutions.

### 2.2 Measurements of routine clinical biochemistry tests

Before and after the hemolysis preparation, all samples received biochemical tests on the analyzer AU5400 (Beckman Coulter) and the Vitros FS 5,1 dry chemistry system (Ortho Clinical Diagnostics). The results were corrected based on the amount of lysed RBCs added. For example, for the 1% hemolysis sample in which 0.01 mL hemolyzed red blood cells were added to 0.99 mL serum, the measured result was divided by 0.99 (99.0%) to adjust the volume dilution.

### 2.3 Data processing and statistical analysis

For each sample, the test results of each analyte at different hemolytic conditions (0%, 0.1%, 0.3%, 1%, 3%, and 10%) were derived by subtracting the original level (the test result at 0% hemolysis). The net results were considered contributions from the hemolyzed RBCs and were summarized in the chart with statistical analyses for use as a reference. For example, the ALT measurements of the first sample after volume dilution correction were 43.10, 43.44, 42.93, 41.11, and 38.14, 29.56 U/L at 0%, 0.10%, 0.30%, 1%, 3%, and 10% hemolysis, respectively. After subtracting 43.10 U/L (0% hemolysis), the results were 0.00, 0.34, −0.17, −1.99, −4.96, −13.54 U/L for summarization in the chart and analyses. The statistical analyses included one‐way ANOVA, linear regression, and correlation coefficient, excluding results beyond the detectable range. *P* < .05 was considered the significant difference.

## 3 RESULTS

**Figure 1:**
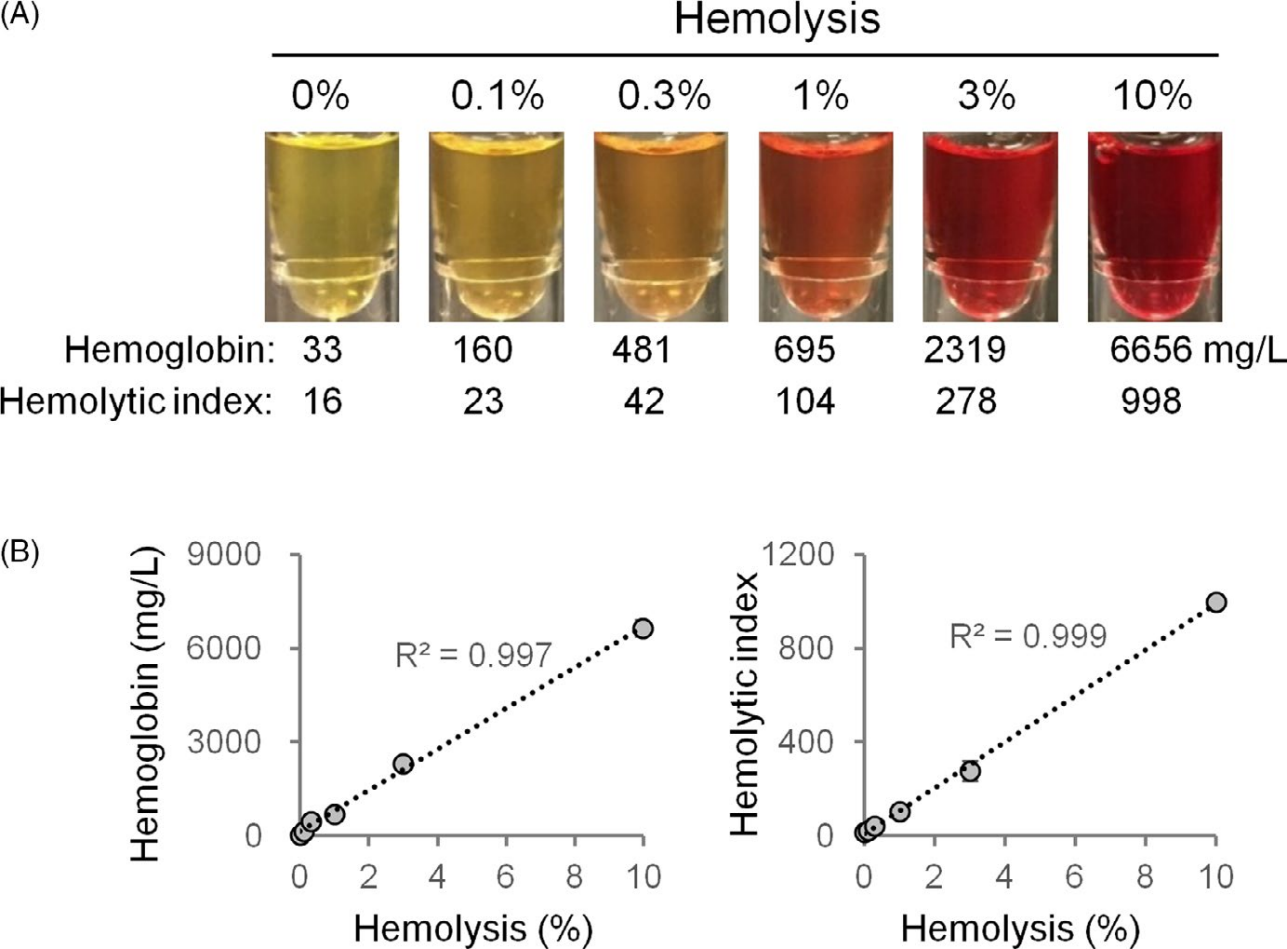
Hemolytic indices following different degrees of hemolysis. A, Demonstration of artificially hemolyzed serum samples. B, Correlations of the hemoglobin concentrations and hemolytic indices with the degrees of hemolysis

In this study, we artificially prepared 0% (original serum without addition of lysed RBCs), 0.1%, 0.3%, 1%, 3%, and 10% hemolyzed serum samples with endogenous RBCs. A visible redness in color was useful for clinical laboratory staff to estimate the extent of hemolysis in a sample and predict how the test results would be affected, allowing them to determine whether a hemolyzed blood sample was acceptable for biochemistry testing. Therefore, we selected these hemolyzed samples for demonstration (Figure 1A).

In these samples, the hemoglobin concentrations were 33, 160, 481, 695, 2319, and 6656 mg/L, and the hemolytic indices were 16, 23, 42, 104, 278, and 998 mg/L, respectively, with both extremely correlating with different degrees of hemolysis (*R* ^2^ = .997 and 0.999, respectively) (Figure 1B).

**Figure 2:**
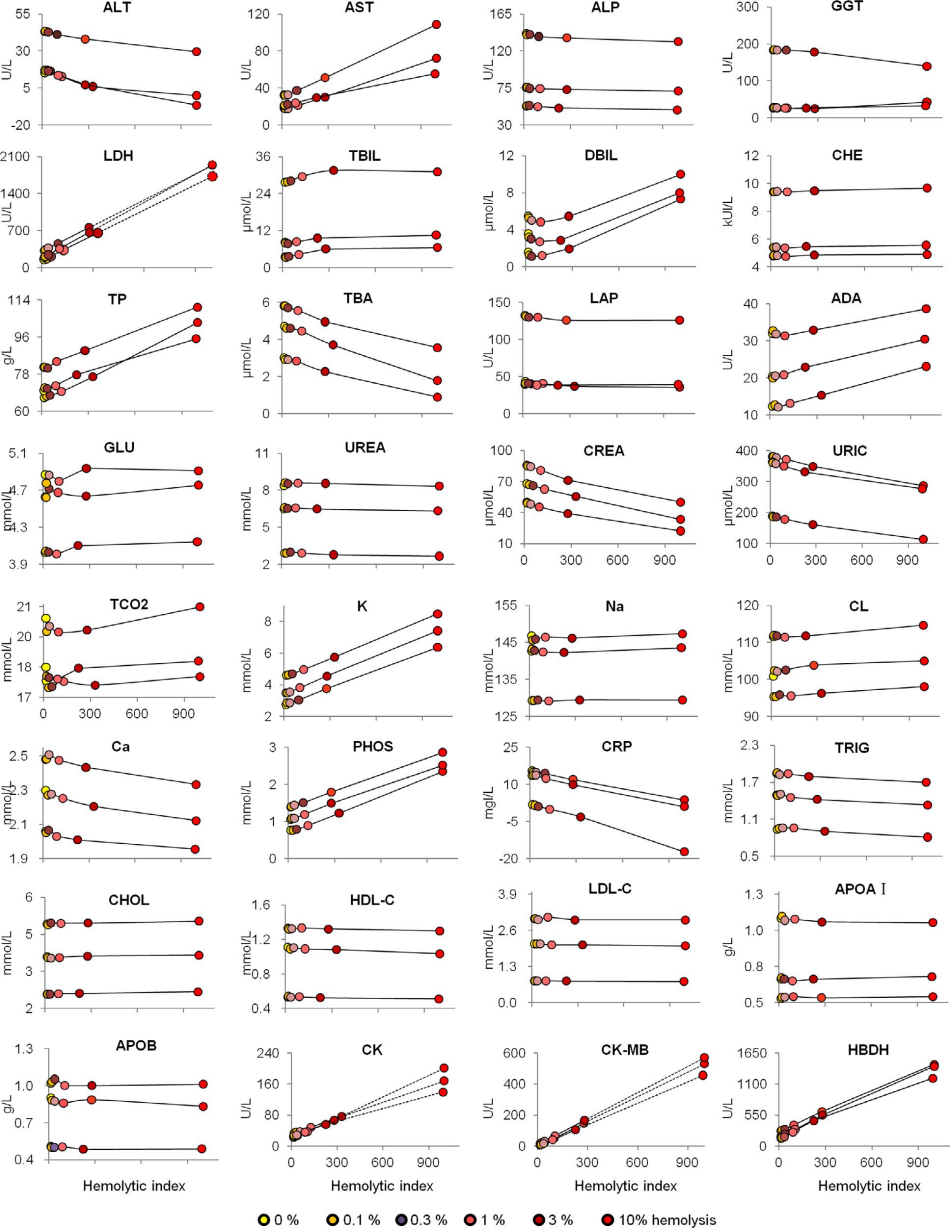
Wet biochemistry results under different hemolysis conditions. Samples from three individuals with results were shown. Dashed points and lines were theoretically derived according to previous results. ALB, albumin; ALT, alanine aminotransferase; AMY; amylase; AST, aspartate aminotransferase; CK, creatinine kinase; CREA, creatinine; GLU, glucose; LDH, lactate dehydrogenase; TBIL, total bilirubin; TP, total protein

**Figure 3:**
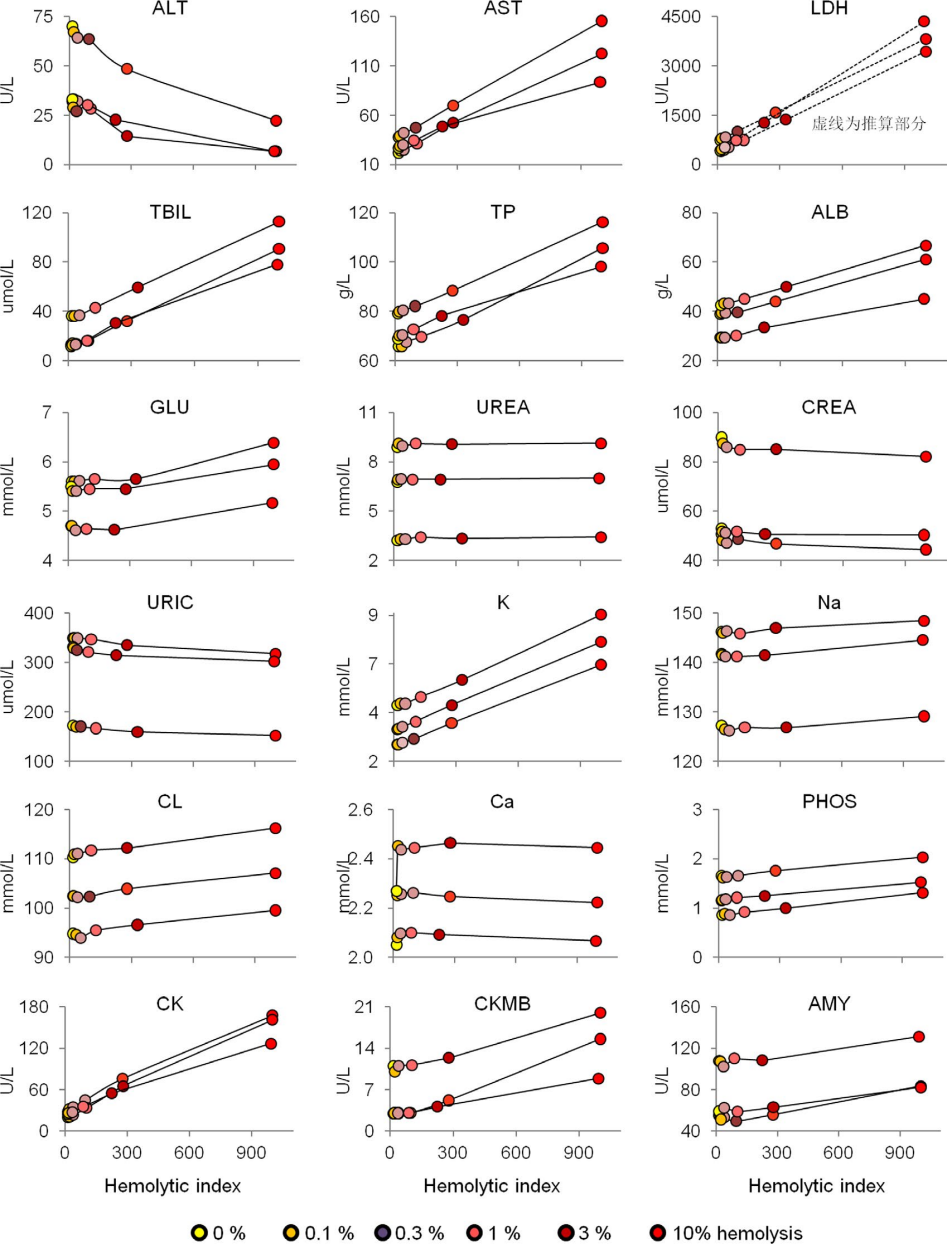
Dry biochemistry results under different hemolysis conditions. Samples from three individuals with results were shown. Dashed points and lines were theoretically derived according to previous results. ALB, albumin; ALT, alanine aminotransferase; AMY; amylase; AST, aspartate aminotransferase; CK, creatinine kinase; CREA, creatinine; GLU, glucose; LDH, lactate dehydrogenase; TBIL, total bilirubin; TP, total protein

All artificially hemolyzed serum samples underwent routine clinical biochemistry tests, including ALT (alanine aminotransferase), AST (aspartate aminotransferase), ALP (alkaline phosphatase), as well as the estimation of 32 other analytes. To simplify the demonstration, the testing results of three individuals are shown in plots (Figure 2). The dry biochemistry test results were also demonstrated (Figure 3).

**Figure 4:**
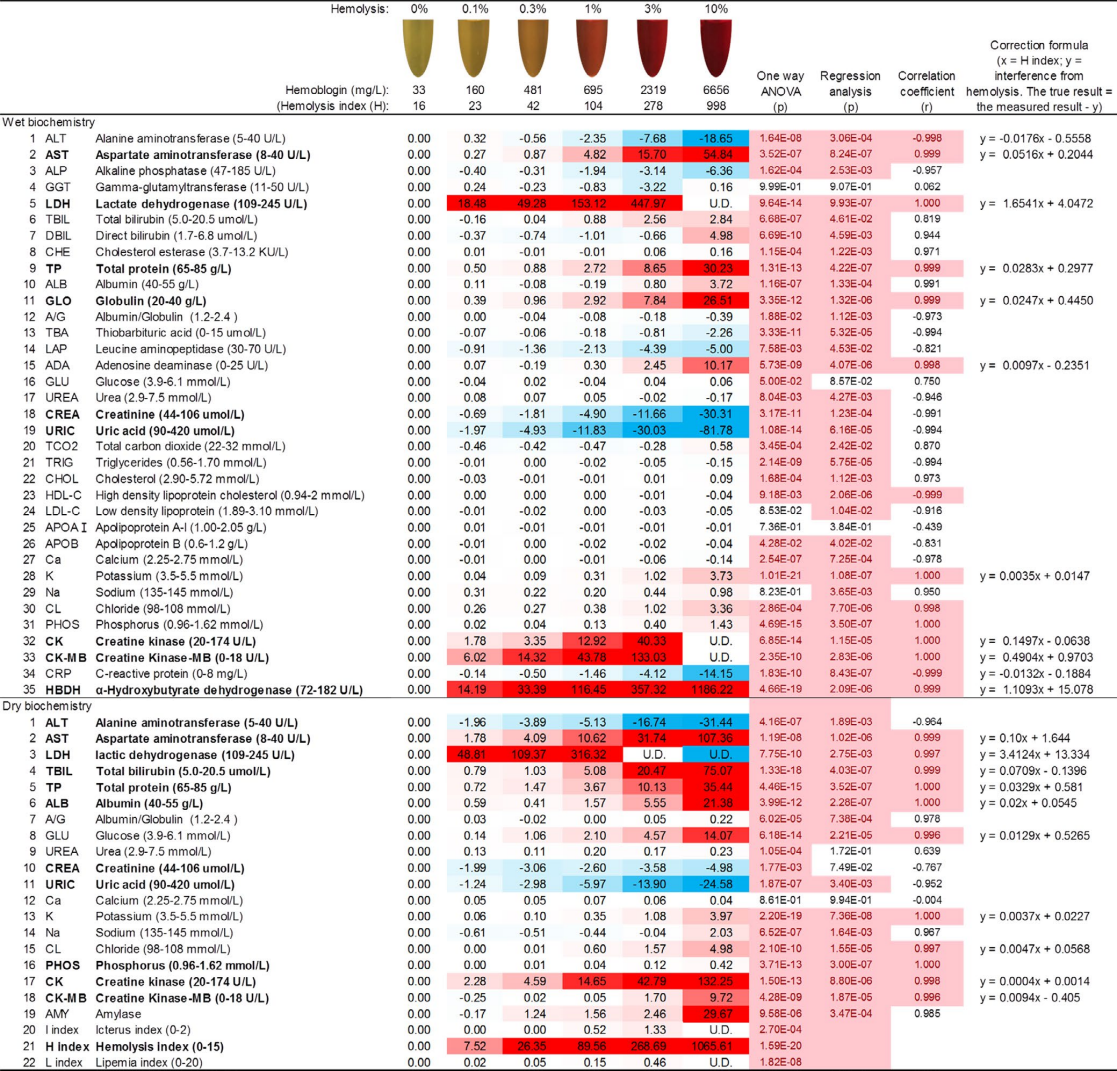
The reference chart of hemolyzed serum samples for clinical chemistry tests. For each analyte, the results of the original sample without the addition of artificially hemolyzed red blood cells (0% hemolysis) were set as the baseline (0.00); the absolute numbers in the table were the increased or decreased part of the results at different hemolysis conditions (0.1%, 0.3%, 1%, 3%, or 10%). The results were the averages of 20 selected samples. One‐way ANOVA (n = 20), linear regression (against H index), and correlation analyses (against H index) were performed (undetectable results were excluded). In the gradient color bars, a half amount of the reference range of each analyte was set for the full colors (eg, for ALT, 22.5 or above was set for the full redness, and −22.5 or below was set for the full blueness). Based on this chart, if a serum sample seems to be about 1% hemolyzed and the ALT test result is 40 U/L, then the true ALT level is likely 37.65 U/L by deducting 2.35 U/L from the test result. “UD” means undetectable. The *P* values smaller than .05 and r values higher than .995 or lower than −.995 were highlighted in red

To provide a visible reference, we summarized all results with the actual appearance of each differentially hemolyzed serum sample into a chart (Figure 4). All test results were adjusted by subtracting the starting result of the original sample (0% hemolysis). The net results at 0.1%, 0.3%, 1%, 3%, and 10% hemolysis were deemed the contribution of the corresponding amount of lysed RBCs. The results of 20 samples were averaged for each analyte and were summarized in a table in the chart, along with statistical analyses. A majority of the analytes showed significant changes (*P* < .05) among the hemolyzed samples, with some heavily affected to the extent that might yield clinically cautious results, such as AST, LDH, TP (total protein), CK, and CK‐MB.

It is well known that the hemolysis index is an extremely useful indicator that quantitatively evaluates the hemolysis of blood cells.^16^, ^17^, ^18^ The hemolytic interference revealed markedly significant regression and very high correlation coefficients with the measured hemolysis indices. For very obvious interference (demonstrated in graded red or blue color bars) with very low statistical p values and high correlations (*r* ≥ .995 or ≤−.995), we also provided a correction formula according to the linear relationship between the interfered results and the hemolysis index values. For example, the *P* values of one‐way ANOVA and regression analyses for ALT were 1.64E‐08 and 3.06E‐04, respectively, and the correlation coefficient of ALT was −.998. If the hemolysis index for a serum sample at about 1% hemolysis is 100 and the result is 40 U/L, then according to the “*y* = −0.0176*x* − 0.5558,” where *x* = H index and *y* = interference from hemolysis, the interference is “−2.316.” Therefore, the true result of this sample is approximately 42.3 U/L. Similar calculations can be performed to derive the true levels of other analytes using the table in the provided chart.

## 4 DISCUSSION

In this study, we provided a reference chart for the quantitative analysis of the impact of hemolysis on routine clinical biochemical tests. Using this chart, clinical laboratory staff can determine whether a hemolyzed serum sample is acceptable and further interpret the results more accurately by removing the interference from hemolysis.

For hemolysis interference, the results of quantitative analysis are largely consistent with previous reports, including changes in LDH, potassium, CK, CK‐MB, and some other analytes.^12^, ^18^, ^19^ However, several previous studies used freeze‐and‐thaw or mechanical trauma without assessing the achievement of cell lysis.^12^, ^16^, ^20^, ^21^ In these studies, the extent of hemolysis was solely determined from the hemolytic index measured by the machine. However, it is impractical for clinical staff to determine whether a clearly hemolyzed blood sample is acceptable because the hemolytic index cannot be obtained until the sample has been analyzed. Furthermore, previous reports have failed to examine the potentially negative effects of freeze‐thaw and mechanical force, which might deactivate the enzymes, leading to the underestimation of analytes. Additionally, some studies have used osmotic shock to lyse red blood cells, which was insufficient to break RBCs completely as the massive release of cellular contents would restore osmolality quickly.

In contrast, we here used gentle sonication to lyse endogenous cells and assessed the completion of cellular lysis, which would provide more analytically accurate analyses. On our chart, the actual color of hemolyzed samples is particularly useful for deciding whether a hemolyzed specimen can be accepted. The correction formulas are especially helpful for hemolyzed samples from patients in an emergent situation when redrawing the blood sample is not feasible. Moreover, in rural or smaller hospitals, clinical chemistry analyzers are not readily available to ascertain the hemolysis index. Therefore, the reference chart is even more significant at these laboratories as they can simply compare the reddish colors of the sample and the standards listed on the chart for estimation.

Reportedly, visual assessment of hemolysis might yield erroneous results and affect patient safety.^22^ However, in this study, the hemoglobin level in the artificially hemolyzed blood sample reached up to 10 g/L, which was much higher than that recorded in the 10% hemolysis sample (hemoglobin: ~7.0 g/L) prepared. In clinics, such heavily hemolyzed blood samples are rare. Based on our chart, most analytes do not change significantly below 1% hemolysis. Therefore, hemolyzed samples presenting a lighter reddish color are acceptable, and the results obtained could be further corrected according to the hemolysis index measured during the analysis.

The interference of hemolysis is considered an additive, which is determined by the number of lysed RBCs, independent of the true level in the original serum. Therefore, the true level can be derived by subtracting the interfering part from the test results. In this study, the table in the chart is a summarization of the results from 20 samples only. Notably, the interference results depend on the amount and composition of RBCs and white blood cells in a particular blood sample analyzed, which might be similar to those of samples recruited for the generation of this chart. Furthermore, we admit that this chart might not apply to samples containing heavy amounts of lipid and high levels of bilirubin. The interference of these analytes and the corresponding correction methodologies could be investigated in future studies.

Herein, we introduced a reference chart for the pre‐analytical screening of hemolytic blood samples in clinical laboratories. By such a chart, hemolyzed samples might still be testable according to the provided correction formula, especially when redrawing blood from the patient is not possible. As hemolysis indices measured using machines from different manufacturers could vary,^23^ individual laboratories could develop similar reference charts based on their clinical biochemistry analyzing systems.
